# Tn antigen promotes human colorectal cancer metastasis via H‐Ras mediated epithelial‐mesenchymal transition activation

**DOI:** 10.1111/jcmm.14117

**Published:** 2019-01-13

**Authors:** Zhe Liu, Jian Liu, Xichen Dong, Xin Hu, Yuliang Jiang, Lina Li, Tan Du, Lei Yang, Tao Wen, Guangyu An, Guosheng Feng

**Affiliations:** ^1^ Department of Oncology Beijing Chao‐Yang Hospital Capital Medical University Beijing China; ^2^ Medical Research Center Beijing Chao‐Yang Hospital Capital Medical University Beijing China

**Keywords:** colorectal cancer, Cosmc, EMT, H‐Ras, metastasis, Tn antigen

## Abstract

Tn antigen is a truncated O‐glycan, frequently detected in colorectal cancer (CRC), but its precise role in CRC metastasis is not well addressed. Here we investigated the effects of Core 1 β3Gal‐T specific molecular chaperone (Cosmc) deletion‐mediated Tn antigen exposure on CRC metastasis and its underlying mechanism. We first used CRISPR/Cas9 technology to knockout Cosmc, which is required for normal O‐glycosylation, and thereby obtained Tn‐positive CRC cells. We then investigated the biological consequences of Tn antigen expression in CRC. The results showed that Tn‐positive cells exhibited an enhanced metastatic capability both in vitro and in vivo. A further analysis indicated that Tn antigen expression induced typical activation of epithelial‐mesenchymal transition (EMT). Mechanistically, we found that H‐Ras, which is known to drive EMT, was markedly up‐regulated in Tn‐positive cells, whereas knockdown of H‐Ras suppressed Tn antigen induced activation of EMT. Furthermore, we confirmed that LS174T cells (Tn‐positive) transfected with wild‐type Cosmc, thus expressing no Tn antigen, had down‐regulation of H‐Ras expression and subsequent inhibition of EMT process. In addition, analysis of 438 samples in TCGA cohort demonstrated that Cosmc expression was reversely correlated with H‐Ras, underscoring the significance of Tn antigen‐H‐Ras signalling in CRC patients. These data demonstrated that Cosmc deletion‐mediated Tn antigen exposure promotes CRC metastasis, which is possibly mediated by H‐Ras‐induced EMT activation.

## 1 INTRODUCTION

Colorectal cancer (CRC) is one of the most common gastrointestinal tumours and the third highest cause of cancer‐related death around the world.^1^, ^2^ Distant metastasis, especially liver metastasis, is the main cause for death in patients with CRC.^3^, ^4^ However, the mechanisms underlying cancer invasion and metastasis are less well defined. More recent studies have shown that abnormal O‐glycosylation of many secreted and membrane‐bound glycoproteins, resulting in expression of the immature truncated O‐glycans such as Tn antigen, is strongly associated with cancer progression and metastasis in various human cancers including CRC.^5^, ^6^, ^7^

Mucin type O‐glycosylation is initiated by addition of N‐acetyl galactosamine (GalNAc) to either serine or threonine, thus forming a biosynthetic intermediate O‐linked structure called Tn antigen.^8^, ^9^ T‐synthase (core 1 β1,3‐galactosyltransferase), a key enzyme in the O‐glycosylation pathway, converts Tn antigen to more complex O‐glycans that eventually modify many secreted and transmembrane glycoproteins.^10^, ^11^ Of note, the expression and activity of T‐synthase require an endoplasmic reticulum (ER)‐resident molecular chaperone Cosmc.^12^, ^13^, ^14^ Dysfunction in T‐synthase or Cosmc leads to the expression of Tn antigen, which is indeed a characteristic of abnormal O‐glycosylation.^15^, ^16^ It has been reported that Tn antigen is detected in almost 90% of human colon cancer whereas it is rarely detected in normal tissue.^15^, ^17^ Tn antigen expression is correlated with tumour invasion and poor prognosis in CRC patients, and it is proposed as a potential target for immunotherapy.^6^, ^18^ Nevertheless, it remains elusive whether Tn antigen may play a causative role in colorectal cancer development and metastasis.

In this study, we forcedly induced Tn antigen expression in both CRC cell lines (HCT116, SW480) through knockout of the Cosmc chaperone. Both in vitro and in vivo experiments showed that Tn antigen directly promoted cancer invasion and metastasis of cells or tumours, suggesting a metastasis‐promoting role for Tn antigen. Moreover, we found that Tn antigen activated the EMT process, which was responsible for the observed oncogenic alterations, by up‐regulating the expression of H‐Ras.

## 2 MATERIALS AND METHODS

### 2.1 Cell culture

Human colorectal cancer cell lines HCT116 and SW480 and human embryonic kidney cells HEK293T were purchased from the American Type Culture Collection (ATCC). The human colorectal cancer cells LS174T (Tn‐positive) were kindly provided by Dr. Tongzhong Ju of the Emory University School of Medicine in Altanta, USA. HCT116 cells were maintained with McCoy's 5A medium (Gibco, Carlsbad, CA, USA). SW480 cells and LS174T cells were maintained with DMEM medium (Sigma, USA). All media contained 10% FBS (Ausbian, Australia) and 1% Penicillin‐Streptomycin solution (Gibco). All cell lines were incubated at 37°C in a humidified atmosphere with 5% CO_2_.

### 2.2 CRISPR/Cas9‐mediated knockout of Cosmc chaperone

The single guide RNA (sgRNA) Oligos targeting Cosmc gene were designed according to the principle of CRISPR/Cas9 system. The sgRNA sequences for human Cosmc exon1 were: F:5′‐CACCGTGTGCTTTGATCACTATGCT‐3′;R:5′‐AAACAGCATAGTGATCAAAGCACAC‐3′. The sgRNA sequences were cloned into linearized LentiCRISPRv2 plasmids, which were co‐transfected into HEK293T cells with psPAX2 and pMD2.G plasmids using Lipofectamine 3000 (Invitrogen, Carlsbad, CA, USA) to produce lentivirus containing CRISPR/Cas9 system targeting Cosmc gene. The virus was transfected into CRC cell lines HCT116 and SW480 with polybrene (Genechem, Shanghai, China). These transfected cells were cultured for 48 h and then selected with 2 μg/mL puromycin.

### 2.3 Flow cytometry

Cells were harvested from the culture flask and resuspended in 100 μL PBS (1 × 10^6^/mL). The mouse anti‐Tn mAb (CA3638, clone 12A8‐C7‐F5, 10 μg/mL, kindly provided by Dr. Tongzhong Ju of the Emory University School of Medicine in Altanta, USA) was used to detect the expression of Tn antigen. After incubation at 4°C for 1 hour, the cells were incubated with PE‐labelled goat antimouse IgM (BD, 562033) for 60 minutes at 4°C. Then the cells were washed twice and then analysed using the flow cytometer (Canto II; BD Bioscience).

### 2.4 Cell migration and invasion assays

After being starved for 24 hours, cells (2 × 10^5^) with serum‐free medium were seeded into the upper chamber of transwell plate (BD Bioscience, 8 μm pore size) pre‐coated with or without Matrigel (BD Bioscience). A 500 μL 10% serum‐containing medium was added to the lower chamber. After being cultured for 24 hours or 48 hours, the migrated or invaded cells were counted under the microscope after fixation with 4% paraformaldehyde and staining with 0.1% crystal violet.

### 2.5 Establishment of transplantable metastatic murine models

The male BALB/c nude mice aged 6 weeks were purchased from Charles River Laboratories (Beijing, China) and maintained under specific pathogen‐free conditions. All animal experiments were performed under the guidelines of Institutional Animal Care and Use Committee at Capital Medical University (Beijing, China) and Medical Research Center of Beijing Chao‐Yang Hospital.

We performed two transplantable metastatic murine models to explore the role of Tn antigen in the metastasis of CRC in vivo. For intrasplenic injection mouse models, 6‐week‐old BALB/c nude mice were anesthetized with chloral hydrate (400 mg/kg) and then incised in the left upper lateral abdomen for 1 cm. The prepared single cell suspension of HCT116 cells with or without Tn antigen expression (2 × 10^6^ per mouse) were injected into the spleen of each mouse (n = 6 for each group) respectively. After 8 weeks, the mice were killed and the livers and lungs were excised and fixed with formalin for H&E staining.

For orthotopic implantation models, 2 × 10^6^ HCT116Tn+ cells or HCT116Tn‐ cells were first injected subcutaneously into 6‐week‐old BALB/c nude mice to attain subcutaneous xenografts that were subsequently minced into 2 mm^3^ pieces and subserosally transplanted into the cecum of other mice (n = 5 for each group). When cachexia occurred, the mice were killed. The xenografts and macroscopically visible metastatic lymph nodes were isolated and embedded in paraffin for histological examinations and immunohistochemistry staining.

### 2.6 Immunohistochemistry

The prepared paraffin‐embedded tissues were cut into 5 μm thick sections that were subsequently dewaxed and rehydrated. After antigen retrieval and blocking endogenous peroxidase, the sections were incubated with specific primary antibody against Tn antigen (2 μg/mL), E‐cadherin (1:500; Proteintech, 20874‐1‐AP) and Snail (1:500, Proteintech, 13099‐1‐AP) at 4°C overnight, followed by incubation with peroxidase labelled secondary antibody at 37°C for 30 minutes. The binding was visualized by the use of DAB reagent (ZSGB‐BIO, China) and the nuclei were counterstained with hematoxylin.

### 2.7 Knockdown of H‐Ras with shRNA

The plasmid containing short hairpin RNA (shRNA) against H‐Ras was purchased from Cyagen Biosciences Inc (ID: VB180523‐1083wxj). The target sequence of the shRNA for human H‐Ras gene is 5′‐CGGAAGCAGGTGGTCATTGAT‐3′. For silencing of H‐Ras in the Tn‐positive cells, transient transfection with the plasmid using Lipofectamine 3000 (Invitrogen) was performed according to the manufacturer's protocol. A vector containing non‐silencing short hairpin RNA (Cyagen Biosciences Inc, China) was used as a control. After 48 hours, the cells were collected for further studies.

### 2.8 Re‐expression of Cosmc in LS174T cells

The GV367‐EGFP‐Cosmc lentiviral particles were produced by Shanghai Genechem Co. Ltd (China) . LS174T cells were transfected with the lentiviral particles with or without a Cosmc gene insert using polybrene (Genechem). The transfected cells were selected using puromycin (2 μg/mL) after 2 days and then pooled for further studies.

### 2.9 RNA extraction and quantitative real‐time PCR

Total RNA was isolated from cells and tissues from xenografts using TRIzol reagent (Invitrogen) according to the manufacturer's instructions. The complementary DNA was synthesized using PrimeScript™ RT Master Mix (TaKaRa, China). The mRNA levels were detected by Real‐time PCR analyses using SYBR Premix (Applied Biosystems) on the 7500 Sequence Detection System (Applied Biosystems) with GAPDH identified as an internal control. The sequences of primers used were shown in Table S1. Relative changes in mRNA were normalized with GAPDH and calculated using 2^‐▵▵CT^ methods.

### 2.10 Western blotting and antibodies

Cells and tissues from xenografts were lysed with RIPA lysis buffer (Solarbio, Shanghai, China) and the protein concentration was determined by BCA assay kit (Thermo Fischer). Equal amount of denatured protein was electrophoresed on 10% SDS‐PAGE and transferred onto PVDF membranes (Millipore). The membranes were blocked with 5% defatted milk and then probed with primary antibodies overnight at 4°C. After incubation of specific HRP‐conjugated secondary antibodies, signals were visualized by ECL kit (Millipore) according to the manufacturer's instruction. Applied antibodies were as follows: Cosmc antibody(1:500; Santa Cruz, sc‐271829), T‐synthase antibody (1:500; Santa Cruz, sc‐100745), H‐Ras antibody (1:200; Santa Cruz, sc‐29), E‐cadherin antibody (1:1000; Cell Signaling Technology, 14472), N‐cadherin antibody (1:1000; Cell Signaling Technology, 13116), Vimentin antibody (1:1000; Cell Signaling Technology, 5741), Snail antibody (1:1000; Cell Signaling Technology, 3879), GAPDH antibody (1:1000; Cell Signaling Technology, 5174).

### 2.11 TCGA colon cancer dataset

To depict the association between Cosmc and H‐Ras in CRC patients, the RNA‐seq data of the 438 cases from TCGA colon cancer dataset were obtained from the cBioPortal for Cancer Genomics (http://www.cbioportal.org/).

### 2.12 Statistical analysis

Each experiment was repeated three times independently to verify reproducibility. Data analysis was performed using the SPSS 22.0 statistical software. The data from in vitro experiments were presented as the mean ± SD. The differences of survival time between the mice bearing Tn‐positive cells and the mice bearing Tn‐negative cells in the orthotopic models was evaluated using a log‐rank test. The none‐parametric Mann‐Whitney *U* test was used when the metastasis rate between two groups was compared. Student's *t* test was used for the statistical analysis. *P *< 0.05 was considered statistically significant.

## 3 RESULTS

### 3.1 Cosmc deficiency induces the expression of Tn antigen in human CRC cell lines

**Figure 1:**
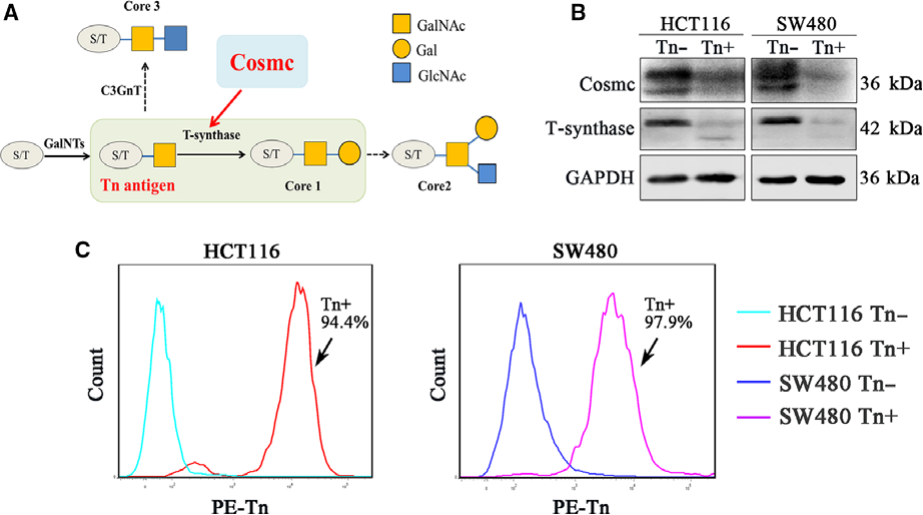
Knockout of Cosmc induced Tn expression in CRC cell lines. A, A chart illustrated the biosynthetic pathway of mucin type O‐glycans and the significant role of Cosmc chaperone in the process of correct O‐glycosylation. B, The knockout of Cosmc chaperone and the degradation of T‐synthase were confirmed by the Western blotting. C, Flow cytometry analysis for the expression of Tn antigen in Cosmc deficiency cells. The percent of Tn positive cells in HCT116 and SW480 cells was 94.4% and 97.9% respectively

To explore the roles of Tn antigen, we first used CRISPR/Cas9 technology to knockout the gene encoding Cosmc, which is specifically required for the process of O‐glycosylation (Figure 1A), in two CRC cell lines (HCT116, SW480). Knockout of Cosmc was confirmed by Western blot analysis (Figure 1B). It showed that T‐synthase was also absent in Cosmc deficiency cancer cells, which was consistent with previous reports that the presence of T‐synthase depends on Cosmc integrity (Figure 1B). Flow cytometry measurement of Tn antigen showed that both CRC cell lines deficiency in Cosmc exhibited an abundant expression of Tn antigen (Tn‐positive), whereas the mock transfected control cells expressed no Tn antigen (Tn‐negative) (Figure 1C).

### 3.2 Tn antigen promotes invasive and metastatic properties of colorectal cancer

**Figure 2:**
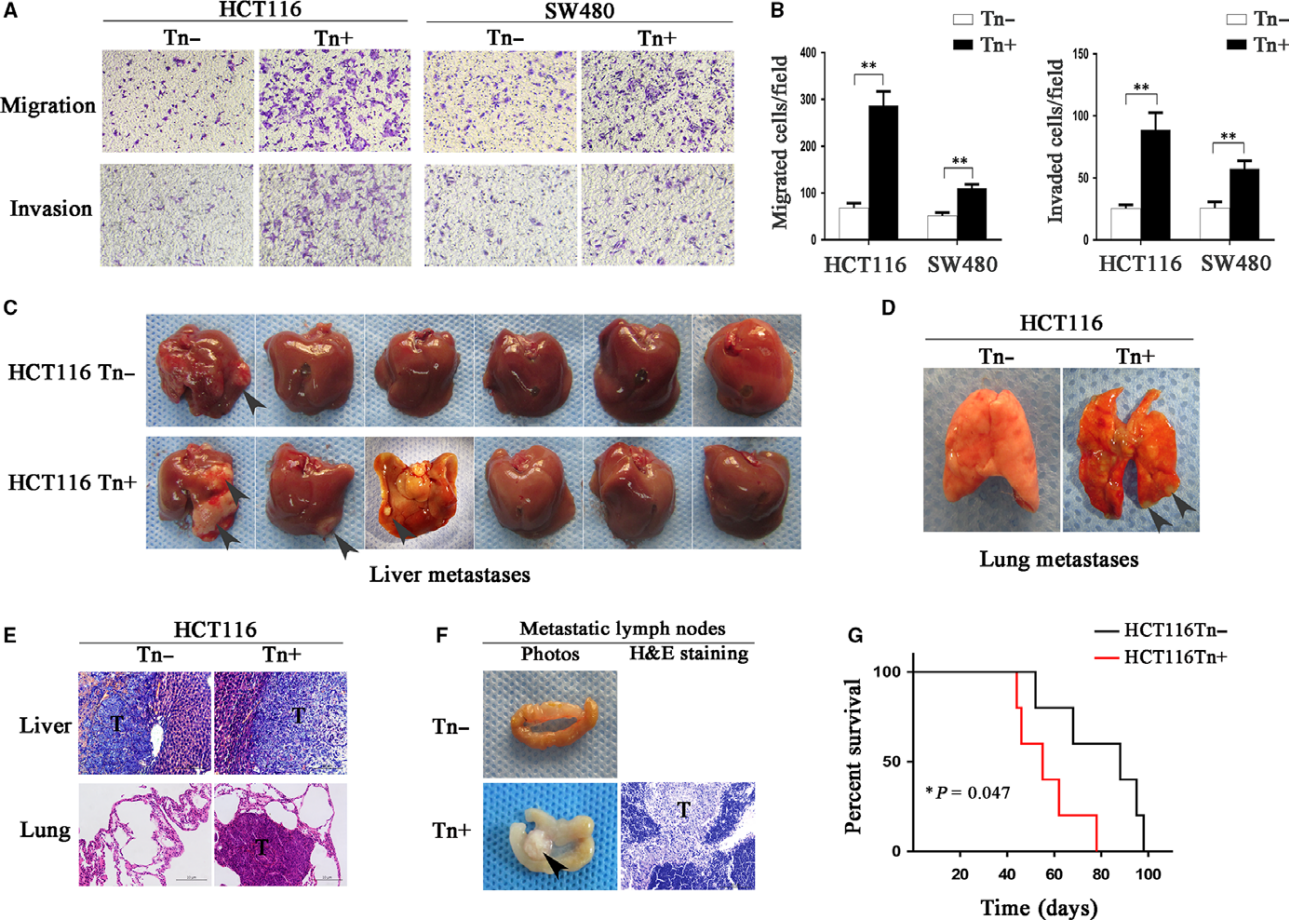
Expression of Tn antigen enhanced the invasive properties. A and B, The prominently increased cell migration and invasion because of the expression of Tn antigen were analysed by transwell assays in both HCT116 and SW480 cells. The indicated migrated and invaded cells were quantified in five randomly chosen fields and presented as mean ± SD (**P < 0.01). C and D, Photographs of liver metastases and lung metastases formation in intrasplenic injection mouse models. E, The formation of liver metastases and lung metastases was confirmed by H&E staining. F, Photographs of metastatic lymph nodes in the mice of orthotopic implantation models. The formation of lymph node metastasis was confirmed by H&E staining. G, Survival rate of the mice in orthotopic implantation models was calculated. The overall survival time in Tn‐positive group was significantly reduced compared with the control (*P < 0.05)

We conducted the transwell assays (matrigel‐uncoated for migration or matrigel‐coated for invasion) to investigate the effects of Tn expression on the invasiveness of CRC cell lines. As shown in Figure 2, both CRC cell lines expressing high levels of Tn antigen (Tn‐positive) displayed drastically enhanced cell migration and invasion compared with the control cells that were Tn‐negative (Figure 2A,B).

As these in vitro experiments indicated that Tn antigen expression was associated with prometastatic traits, we next tested whether Tn antigen could promote metastasis in vivo. Here, we intrasplenically injected the HCT116 cells expressing Tn antigen (Tn‐positive) as well as their corresponding control cells (Tn‐negative) to establish a metastatic colorectal cancer mouse model. After an 8‐week inoculation, we observed that three of the six mice bearing Tn‐positive cell tumours formed liver metastases (3/6, 50.0%), whereas only one of six mice transplanted with Tn‐negative cells was found with liver metastasis (1/6, 16.7%) (Figure 2C). Besides, one mouse injected with Tn‐positive cells displayed pulmonary metastasis (1/6, 16.7%), which represented a more severe outcome, but no pulmonary metastasis was found in the Tn‐negative group (0/6) (Figure 2D). H&E staining analysis confirmed the existence of tumours in the liver or lung metastases (Figure 2E).

We also established an orthotopic implantation murine model, which ideally mimics the in vivo invasion and metastasis of cancer, to investigate the effects of Tn antigen on CRC metastasis.^19^, ^20^ Although there was no liver or lung metastasis detected in the mice undergoing orthotopic transplantation, we still observed that the mice implanted with Tn‐positive cells presented with more lymph node metastases compared with those implanted with Tn‐negative cells (5/5, 100% vs 3/5, 60%) (Figure 2F and Figure S1). Besides, Tn antigen led to a significantly reduced survival time in the mice bearing Tn‐positive xenotransplants (*P* = 0.047) (Figure 2G and Table S2). Collectively, Tn antigen expression significantly enhanced the in vivo metastasis of CRC (9 of 11 vs 4 of 11, *P* = 0.034, Table 1), and resulted in a worse prognosis.

**Table 1:** The metastatic rate in both transplantable metastatic murine models

Groups	Intrasplenic implantation mouse model^a^		Orthotopic implantation mouse model^a^	Metastatic Rate^b^	*P*‐value^b^
	Liver metastasis	Lung metastasis	Lymph node metastasis		
Tn+	3/6	1/6	5/5	9/11	P* = 0.034
Tn‐	1/6	0/6	3/5	4/11	

a Distant metastases to specific organs or regional lymph nodes occurred in both mouse models.

b There was a significantly increased incidence of metastasis in Tn‐positive cells implanted mice. (9 of 11 vs 4 of 11, P* < 0.05).

### 3.3 Tn antigen promotes CRC invasiveness and migration by activating EMT

**Figure 3:**
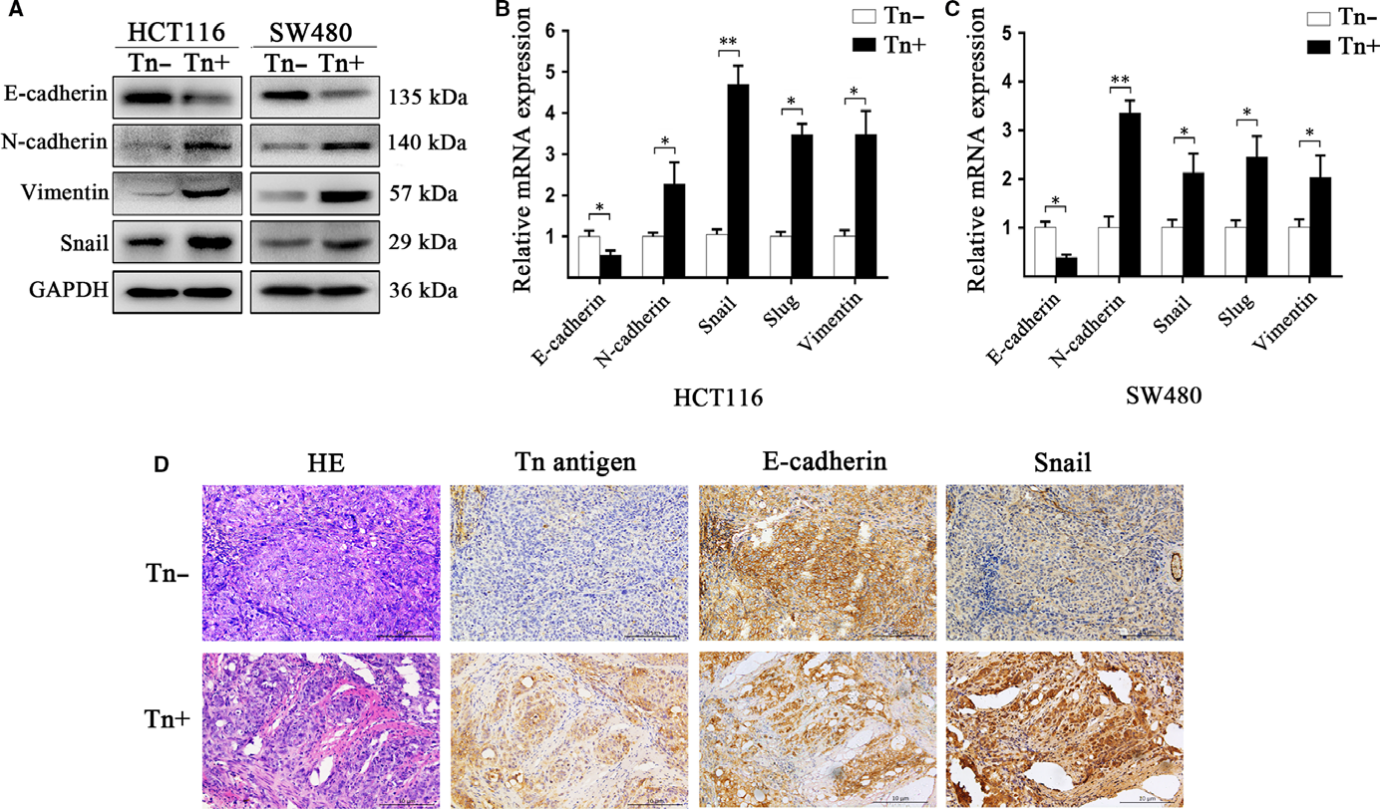
Expression of Tn antigen in human colorectal cancer promoted EMT. A, The expression of E‐cadherin, N‐cadherin, Vimentin and Snail in HCT116 and SW480 cells were analysed by western blotting. B and C, Relative mRNA expression of E‐cadherin, N‐cadherin, Snail, Slug and Vimentin were calculated by qRT‐PCR in HCT116 and SW480 cells. (*P < 0.05 and **P < 0.01). D, Immunohistochemistry staining in the xenografts in orthotopic implantation murine models showed that there was a reduced E‐cadherin expression but an increased Snail expression in Tn‐positive group

**Figure 4:**
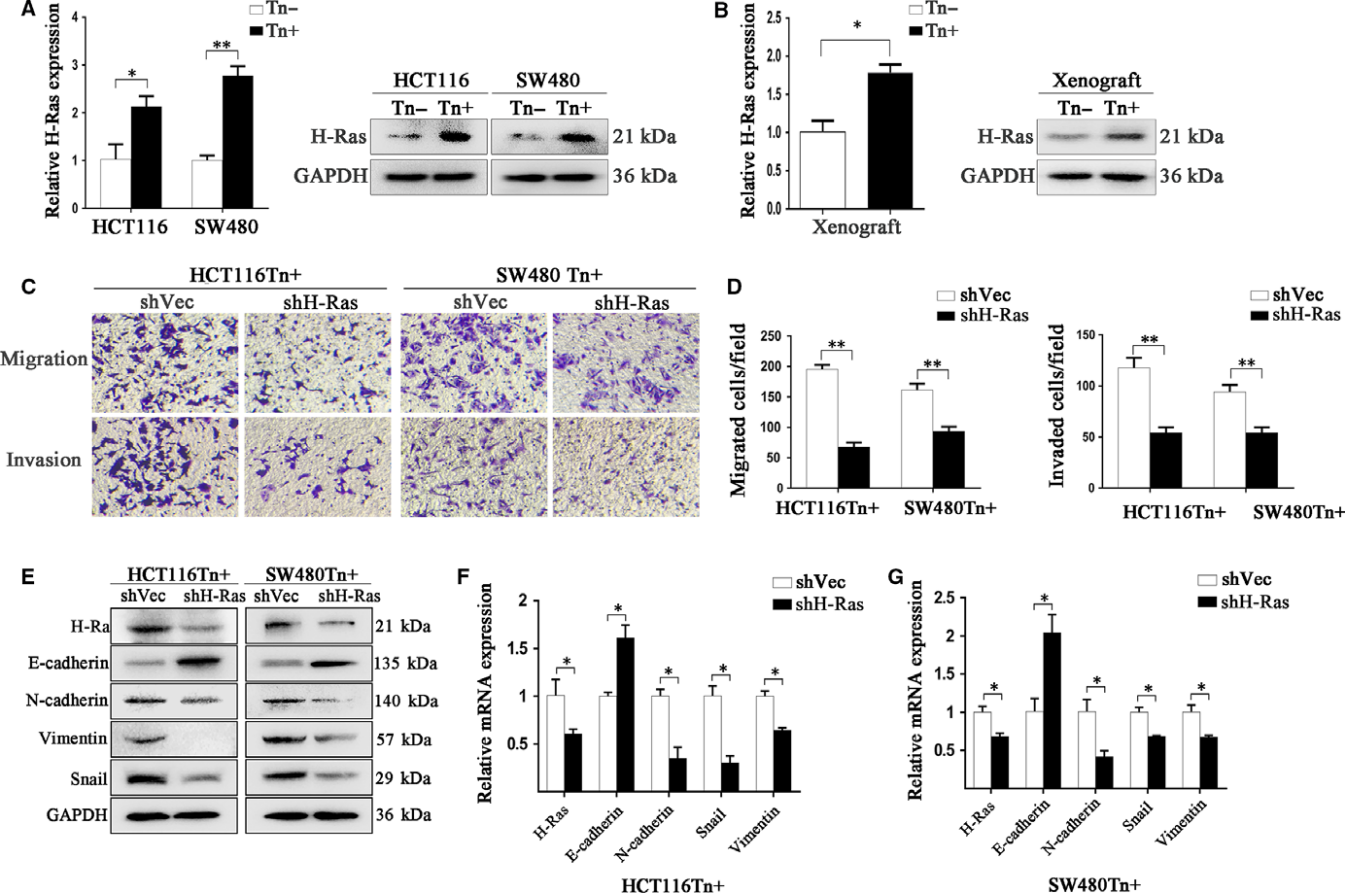
Increased H‐Ras expression contributed to the activated EMT in Tn‐positive cells. A and B, The significantly increased H‐Ras expression in Tn‐positive CRC cells and Tn‐ positive xenografts was confirmed by Real‐time PCR and Western blotting (*P < 0.05 and **P < 0.01). C and D, The ability of cell migration and invasion in Tn positive cells with H‐Ras knockdown and the control was analysed by transwell assays. Significantly reduced cell migration and invasion was detected after down‐regulation of H‐Ras in both HCT116Tn+ and SW480Tn+ cells (**P < 0.01). E and G, The influence of H‐Ras down‐regulation on the levels of E‐cadherin, N‐cadherin, Vimentin and Snail in both Tn‐positive cells was analysed by Western blot and Real‐time PCR (*P < 0.05)

Next, we asked how Tn antigen plays a metastasis‐promoting role in CRC. In light of the critical role of the EMT process in tumourigenesis and metastasis,^21^, ^22^, ^23^ we examined whether Tn antigen activates EMT pathway. As shown in Figure 4, both CRC cell lines expressing Tn antigen demonstrated typical EMT characteristics, evidenced by a significant decrease in the expression of E‐cadherin, a canonical epithelia marker, and an enhancement in several mesenchymal markers such as N‐cadherin, Vimentin and Snail. The changes of these EMT‐associated markers were similar in either protein or mRNA level (Figure 3A‐C). Concurrently, we used immunohistochemical staining to detect the EMT‐associated markers in the xenografts from the metastatic colorectal cancer murine models. As expected, Tn‐positive xenografts showed a decreased E‐cadherin expression and an increased expression of Snail, suggesting an occurrence of the EMT process in vivo (Figure 3D). Together, these data indicated that Tn antigen expression was able to activate the EMT process in CRC.

### 3.4 Tn antigen targets H‐Ras to activate the EMT process

We further investigated the mechanisms by which Tn antigen activates EMT. Accumulating evidence shows that Ras signalling activates EMT during cancer metastasis.^24^, ^25^ We reasoned whether Tn antigen has an influence on Ras family proteins (H‐Ras, K‐Ras and N‐Ras), and consequently, activates EMT. We found that H‐Ras, but not K‐ras or N‐ras, was prominently up‐regulated in both Tn‐positive CRC cells as compared with that in Tn‐negative cells (Figure 4A and Figure S2). We next detected H‐Ras expression in the xenografts from the metastatic colorectal cancer murine models. As expected, Tn‐positive xenografts exhibited markedly up‐regulated H‐Ras expression (Figure 4B), suggesting that Tn antigen may activate EMT via H‐Ras.

To validate the role of H‐Ras in Tn antigen‐induced EMT, we knocked down H‐Ras expression in both Tn‐positive cell lines with shRNA, and found that H‐Ras knockdown markedly reduced Tn antigen‐induced cell migration and invasion in both HCT116 and SW480 cells (Figure 4C,D). We also examined the expression levels of EMT‐associated markers and found that H‐Ras knockdown largely recovered the expression of E‐cadherin and decreased the expression of N‐cadherin, Vimentin and Snail, indicating that H‐Ras knockdown reversed Tn‐induced EMT (Figure 4E‐G). Together, these data suggest that H‐Ras was likely responsible for Tn antigen‐induced metastatic properties enhancement and EMT activation.

### 3.5 Re‐expression of Cosmc in LS174T cells (Tn‐positive) reduces H‐Ras expression and subsequent activation of EMT

**Figure 5:**
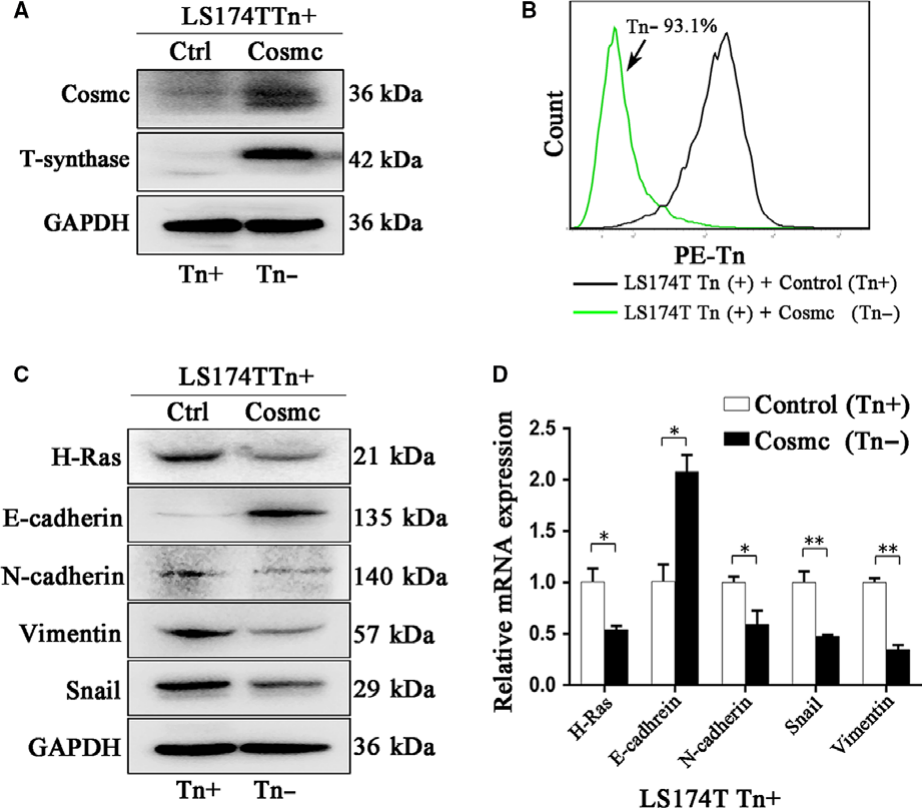
Re‐expression of Cosmc in Tn positive cells down‐regulated the expression of H‐Ras. A, Re‐expression of Cosmc and the consequent restoration of T‐synthase in LS174TTn(+) cell were confirmed by Western blotting. B, The expression of Tn antigen in LS174TTn(+) cells transfected with wide‐type Cosmc was detected by FACS. C, Reduced H‐Ras expression was detected by western blotting in the transfected Tn positive cells together with the increased expression of E‐cad and reduced expression of N‐cadherin, Snail and Vimentin in protein level. D, Decreased mRNA expression of H‐Ras and corresponding changes of reversed EMT were analysed by Real‐time PCR in the LS174TTn(+) cells transfected with COSMC compared with the control cells (*P < 0.05 and **P < 0.01)

To further confirm the above results, we included another colorectal carcinoma cell line, LS174T (Tn‐positive). This cell line harbours mutated Cosmc that encodes a dysfunctional Cosmc leading to an inactive T‐synthase and resultant Tn antigen expression.^26^ After stable transfection with wild‐type Cosmc (Figure 5A), the Tn antigen was abolished in LS174T cells (Tn‐negative) (Figure 5B). As a consequence, we found that the expression level of H‐Ras was much reduced in LS174T cells transfected with Cosmc (Tn‐negative), as compared with that in LS174T transfected with blank vector (Tn‐positive). Further analyses on the activation of the EMT process confirmed that loss of Tn antigen suppressed the EMT characteristics, evidenced by an enhancement of epithelial marker, E‐cadherin and a decrease in mesenchymal markers such as N‐cadherin, Snail and Vimentin (Figure 5C,D).

### 3.6 Determination of Tn antigen‐H‐Ras signalling in patients

**Figure 6:**
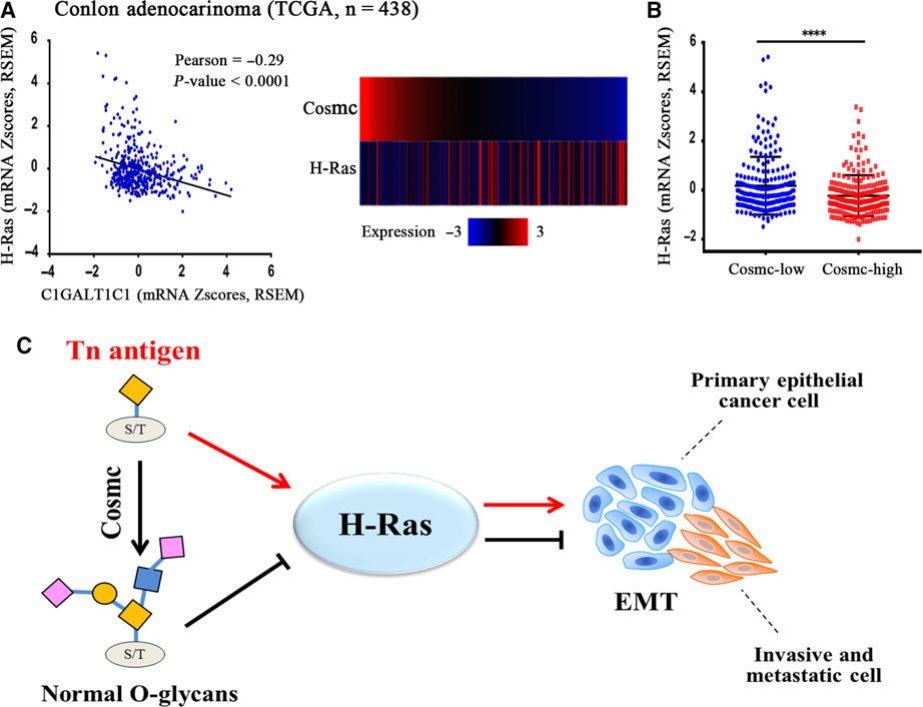
The detection of Tn antigen‐H‐Ras‐EMT signalling in CRC patients. A and B, The bioinformatics analysis using transcriptome data of 438 CRC patients in TCGA cohort revealed that Cosmc expression was negatively correlated with H‐Ras expression at mRNA level (*P* < 0.0001). C, Tn antigen targets H‐Ras to promote EMT in colorectal cancer. We proposed the hypothesis that expression of Tn antigen increases H‐Ras expression, which contributes to the intensive activation of EMT and consequently potentiates the malignant properties in colorectal cancer

As these experiments indicated that Tn antigen expression was associated with H‐Ras mediated EMT, we further investigated 438 cases in the TCGA cohort to delineate the relationship between Tn antigen and H‐Ras in patients. Because expression of Tn antigen was induced by Cosmc deficiency, we performed a correlation analysis between Cosmc and H‐Ras using the transcriptome data of colon cancer samples in TCGA. As it can be seen, Cosmc expression levels showed a significant reverse correlation with H‐Ras levels (*r* = −0.29, *P* < 0.0001, Figure 6A) in the colon cancer tissues. The expression levels of H‐Ras in Cosmc‐low patients were significantly higher compared with the Cosmc‐high patients (*P* < 0.0001, Figure 6B). All these data were consisted with the above in vitro and in vivo results and highlighted the significance of Tn antigen‐H‐Ras signalling in patients.

## 4 DISCUSSION

In this study, we investigated the pathological role of Tn antigen in colorectal cancer metastasis using in vitro and in vivo experiments. We used precise gene editing to knockout the Cosmc chaperone to induce the expression of Tn antigen in human colorectal cancer cells HCT116, SW480. We showed that Tn antigen contributed to the increased invasive and metastatic properties of CRC in vitro and in vivo and revealed that up‐regulation of H‐Ras expression and subsequent activation of EMT could be a primary mechanism responsible for these observed oncogenic features. We further demonstrated a negative relationship between Cosmc and H‐Ras via analysis of the transcriptome data in the TCGA cohort, indicating the importance of the Tn antigen‐H‐Ras signalling in CRC patients. Our results thereby present a new insight into how Tn antigen promotes colorectal cancer metastasis.

It has been well documented that Tn antigen is one of the most expressed tumour antigen found in many human cancers, such as colon,^27^ breast,^28^ cervix,^29^ stomach^30^ and skin.^31^ Many studies have established an association of Tn antigen expression with cancer progression and metastasis and poor outcome.^13^, ^32^, ^33^ However, it is still unclear whether Tn antigen plays a causative role in cancer progression and metastasis. There are indeed some controversies regarding the biological consequences of Tn antigen in tumourigenesis. Most researches supported a tumour‐promoting role for Tn antigen. For instance, Bapu et al showed that the expression of Tn antigen in breast cancer contributes to specific adhesive interactions between cancer cells and endothelium, which promotes tumour metastasis and poor progression.^34^ Hofmann et al demonstrated that forced expression of Tn antigen in pancreatic cancer cells leads to increased migration and decreased proliferation and apoptosis.^35^ Lin et al also found that Tn expression in oral squamous cell carcinoma enhances the invasive potential of tumour cells through up‐regulating EGFR phosphorylation and activity.^36^ Conversely, Bergstrom and colleagues unexpectedly found that Tn antigen is not involved with cancer progression in a murine model of colorectal cancer; instead intestinal inflammation is a major mechanism responsible for tumour development.^37^ Song et al even reported that Tn antigen expression suppressed breast cancer development in mice via impairment of MUC1 expression, which is usually highly expressed in various cancers.^38^ All of these findings suggest that the role of Tn antigen in cancer progression and metastasis needs to be further understood.

In the process of O‐glycosylation, the Tn antigen is normally modified to form elongated and complex O‐glycans by a specific galactosyltransferase (T‐synthase) in the Golgi apparatus of cells and is thereby not detectable in normal tissues.^14^, ^39^ T‐synthase and its unique chaperone Cosmc are indispensable for normal O‐glycosylation.^13^, ^40^ As many studies revealed that dysfunction in Cosmc (mutations, deletion, and hypermethylation) is a prevailing mechanism for Tn antigen expression in various human cancers,^7^, ^26^, ^35^, ^41^ we focused our attention on the functional role of Tn antigen by induction of Cosmc deficiency. Here we depleted the Cosmc chaperone using CRISPR/Cas9 system in both CRC cell lines and induced abundant Tn antigen expression in cells. We therefore acquired CRC cells carrying Tn antigen (Tn‐positive) versus Tn‐negative control cells, which enabled us to analyse how Tn antigen contributes to CRC progression and metastasis.

Our results clearly showed that both CRC cells expressing Tn antigen enhanced the capabilities of cell migration and invasion in vitro. More importantly, two types of transplantation metastatic mouse models (intrasplenic injection model and orthotopical implantation model^42^, ^43^) confirmed that tumour metastasis was more evident in Tn‐positive group compared with the control mice expressing no Tn antigen. These findings provide direct evidence that Tn antigen promotes CRC metastasis, which may be considered as a target for immunotherapy in future.

We next asked how Tn antigen affects cancer metastasis in CRC. Because of tumour metastasis is a complicated and multi‐step course in which EMT has been considered to be associated with the increased invasive and metastatic properties,^44^, ^45^, ^46^ we assumed that activation of EMT may play an important role in Tn antigen‐induced metastasis. We found that Tn antigen significantly activated the EMT pathway, demonstrated by a reduced expression of epithelial cell marker such as E‐cadherin, and an enhanced expression of mesenchymal cell markers with both in vitro and in vivo assays. We thereby concluded that Tn antigen promotes cancer metastasis through activation of the EMT pathway. We further explored the mechanism as to how Tn antigen activates EMT.

More recently, it has been reported that Ras/MAPK signalling pathway is responsible for activation of EMT, and is associated with tumourigenesis.^24^, ^47^, ^48^ Suppression of Ras signalling by Rnd1 inhibited the following signalling cascades and consequently deregulated the activation of EMT.^49^ We checked the expression profiles of Ras genes (H‐Ras, K‐Ras, N‐Ras) in Tn‐positive vs Tn‐negative CRC cells, and found an increased expression of H‐Ras in cancer cells expressing Tn antigen, whereas the changes of K‐Ras or N‐Ras were not different. Several studies revealed that ectopic expression of oncogenic H‐Ras led to an EMT and increased invasive ability, which were indeed consistent with our observations.^25^, ^50^ We found that knockdown of H‐Ras resulted in a suppression of EMT and reduced the ability of cell migration and invasion, suggesting that H‐Ras could be a key factor responsible for Tn antigen‐induced cancer metastasis. This observation was confirmed by transfection of wt Cosmc into LS174T cells to abolish Tn antigen expression, which further resulted in a decreased expression of H‐Ras and the reduced EMT characteristics. Furthermore, the reverse association detected between Cosmc and H‐Ras expression in 438 colon patients in TCGA cohort suggested that Tn antigen/H‐Ras signalling occurred extensively in patients, which was in accordant with our in vivo and in vitro investigations.

In summary, our data showed that Tn antigen expression, a hallmark of abnormal O‐glycosylation, may contribute to colon cancer metastasis. Tn antigen may promote activation of the EMT process by up‐regulating the expression of H‐Ras, as summarized in our model (Figure 6C). Our study underscored the importance of Tn antigen in CRC progression and metastasis, and suggested that anti‐Tn antigen may hold a great promise for tumour immunotherapy.

## CONFLICT OF INTEREST

The authors declare that they have no competing interests.
