# Supplementary figures and images for: Tn antigen promotes human colorectal cancer metastasis via H‐Ras mediated epithelial‐mesenchymal transition activation
Source: J Cell Mol Med. 2019 Jan 13;23(3):2083–92. doi: 10.1111/jcmm.14117 (PMC6378212; doi:10.1111/jcmm.14117)

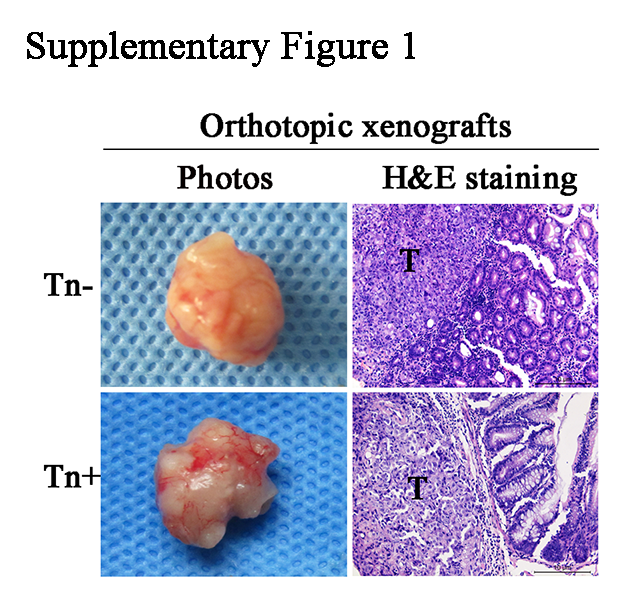

Supplement: Supplementary file 1 [file JCMM-23-2083-s001.tif]

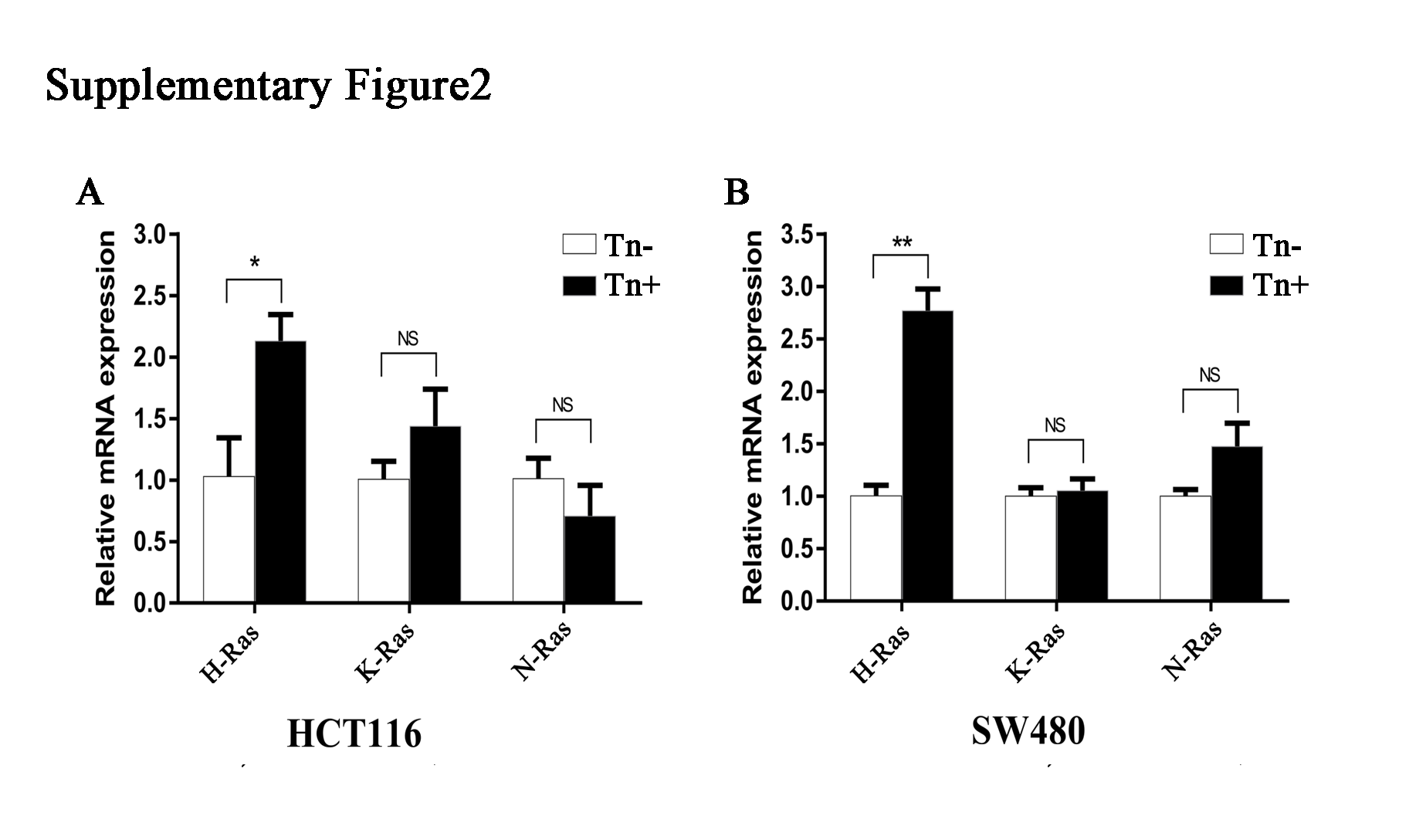

Supplement: Supplementary file 2 [file JCMM-23-2083-s002.tif]
