# Supplementary material for: Tn antigen promotes human colorectal cancer metastasis via H‐Ras mediated epithelial‐mesenchymal transition activation
Source: J Cell Mol Med. 2019 Jan 13;23(3):2083–92. doi: 10.1111/jcmm.14117 (PMC6378212; doi:10.1111/jcmm.14117)
Supplement: Supplementary file 3 [file JCMM-23-2083-s003.doc]

| **Gene** | **Primer sequences(5’-3’)** |
| --- | --- |
| E-cadherin | F: CGAGAGCTACACGTTCACGG  R: GGGTGTCGAGGGAAAAATAGG |
| N-cadherin | F: TCAGGCGTCTGTAGAGGCTT  R: ATGCACATCCTTCGATAAGACTG |
| Snail | F: AAGGCCTTCTCTAGGCCCT  R: CGCAGGTTGGAGCGGTCAG |
| Slug | F: CGAACTGGACACACATACAGTG  R: CTGAGGATCTCTGGTTGTGGT |
| Vimentin | F: AGTCCACTGAGTACCGGAGAC  R: CATTTCACGCATCTGGCGTTC |
| H-Ras | F: ATGACGGAATATAAGCTGGTGGT  R: GGCACGTCTCCCCATCAATG |
| K-Ras | F: GGACTGGGGAGGGCTTTCT  R: GCCTGTTTTGTGTCTACTGTTCT |
| N-Ras | F: CAGGGAGCAGATTAAGCGAGT  R: GGGCTTGTTTTGTATCAACTGTC |
| GAPDH | F: AATCCCATCACCATCTTCCA  R: TGGACTCCACGACGTACTCA |

Supplementary Table 1. The sequences of primers used in our experiments
