# Supplementary material for: Tn antigen promotes human colorectal cancer metastasis via H‐Ras mediated epithelial‐mesenchymal transition activation
Source: J Cell Mol Med. 2019 Jan 13;23(3):2083–92. doi: 10.1111/jcmm.14117 (PMC6378212; doi:10.1111/jcmm.14117)
Supplement: Supplementary file 4 [file JCMM-23-2083-s004.doc]

Supplementary Table 2.The survival time of each mouse implanted with Tn-positive cells and Tn-negative cells in orthotopic mouse models.

| **Number** | **Survival time(days)** | |
| --- | --- | --- |
|  | HCT116 (Tn-) | HCT116 (Tn+) |
| 1 | 52 | 44 |
| 2 | 68 | 46 |
| 3 | 88 | 55 |
| 4 | 95 | 62 |
| 5 | 98 | 78 |
