# Supplementary material for: Tn antigen promotes human colorectal cancer metastasis via H‐Ras mediated epithelial‐mesenchymal transition activation
Source: J Cell Mol Med. 2019 Jan 13;23(3):2083–92. doi: 10.1111/jcmm.14117 (PMC6378212; doi:10.1111/jcmm.14117)
Supplement: Supplementary file 5 [file JCMM-23-2083-s005.doc]

**Supplementary Figure Legends**

**Supplementary Figure 1.** Xenografts were observed in orthotopic implantation mouse models. The formation of orthotopic xenografts was confirmed by H&E staining

**Supplementary Figure 2.** Expression of H-Ras, but not K-Ras or N-Ras, was significantly increased in Tn-positive cells. (A and B) The mRNA expression of H-Ras, K-Ras and N-Ras in Tn-positive cells as well as the control cells was analyzed by Real-time PCR. Results showed that there was an obviously increase in the expression of H-Ras, while there was no significant difference in K-Ras or N-Ras.
